# Ensemble Learning-Based Pulse Signal Recognition: Classification Model Development Study

**DOI:** 10.2196/28039

**Published:** 2021-10-21

**Authors:** Jianjun Yan, Xianglei Cai, Songye Chen, Rui Guo, Haixia Yan, Yiqin Wang

**Affiliations:** 1 Institute of Intelligent Perception and Diagnosis School of Mechanical and Power Engineering East China University of Science and Technology Shanghai China; 2 Shanghai Key Laboratory of Health Identification and Assessment Laboratory of Traditional Chinese Medicine for Diagnostic Information Shanghai University of Traditional Chinese Medicine Shanghai China

**Keywords:** wrist pulse, ensemble learning, support vector machine, deep convolutional neural network, pulse signal, machine learning, traditional Chinese medicine, pulse classification, pulse analysis, fully connected neural network, synthetic minority oversampling technique, feature extraction

## Abstract

**Background:**

In pulse signal analysis and identification, time domain and time frequency domain analysis methods can obtain interpretable structured data and build classification models using traditional machine learning methods. Unstructured data, such as pulse signals, contain rich information about the state of the cardiovascular system, and local features of unstructured data can be extracted and classified using deep learning.

**Objective:**

The objective of this paper was to comprehensively use machine learning and deep learning classification methods to fully exploit the information about pulse signals.

**Methods:**

Structured data were obtained by using time domain and time frequency domain analysis methods. A classification model was built using a support vector machine (SVM), a deep convolutional neural network (DCNN) kernel was used to extract local features of the unstructured data, and the stacking method was used to fuse the above classification results for decision making.

**Results:**

The highest average accuracy of 0.7914 was obtained using only a single classifier, while the average accuracy obtained using the ensemble learning approach was 0.8330.

**Conclusions:**

Ensemble learning can effectively use information from structured and unstructured data to improve classification accuracy through decision-level fusion. This study provides a new idea and method for pulse signal classification, which is of practical value for pulse diagnosis objectification.

## Introduction

A pulse signal contains a large amount of pathological and physiological information [1,2], and the signal characteristics are closely related to diseases (hypertension, atherosclerosis, etc), especially cardiovascular disease (CVD) and physiological parameters (pulse wave velocity, blood pressure, etc) [3,4]. Therefore, pulse analysis is widely used for cardiovascular function assessment and noninvasive early diagnosis of CVD and related complications [5]. It is a convenient, noninvasive, and effective diagnostic method that is widely used in traditional Chinese medicine (TCM). In recent years, smart wearable devices have become increasingly popular, allowing individuals to monitor their own pulse status. However, the important information contained in the pulse signal requires a highly experienced TCM practitioner to make a diagnosis, which is highly variable [6]. In Chinese medicine, pulses are classified into 28 single-pulse types based on 4 major elements: pulse depth, pulse rate, pulse shape, and pulse intensity [6,7]. A patient's pulse may be a combination of several single-pulse types, that is, a compound pulse [8], and a compound pulse may carry more physiological information and be more difficult to distinguish and identify. For example, a slippery pulse and a flat pulse are the main pulse types in healthy people; a thready pulse may be due to overexertion and deficiency of qi and blood; a stringy pulse may be related to liver disorders; a thready, slippery pulse may be related to colds; a thready, stringy pulse may be related to kidney disorders; and a stringy, slippery pulse may be related to coughing, dizziness, and weakness.

Practitioners of Chinese medicine make a diagnosis by touching the patient's wrist and feeling the patient's pulse with their fingers for several minutes to determine the patient's pulse type through experience and make medical decisions accordingly.

Using deep learning or machine learning methods, pulse types can be better classified to help medical practitioners with diagnosis. For individuals, without medical background and experience, the pulse types obtained can also be collected and analyzed by wearable devices to obtain a preliminary understanding of their physical condition and can better prevent CVD. There is already a good deal of scholarly research related to the classification of pulse types. Xu et al [9] used Lempel-Ziv complexity analysis to detect arrhythmic pulses. This approach was applied on 140 clinic pulses for detecting 7 pulse patterns. Zhang et al [10] referred to the edit distance with real penalty (ERP) and the progress in k-nearest-neighbor (KNN) classifiers using an ERP-based KNN classifier on the classification of pulse waveforms. Garmaev et al [11] used cluster analysis of the time parameters of a pulse signal to classify pulses. After clustering, the data were evaluated using the nonparametric Kruskel-Wallis test. Li et al [12] used five CVD and complications extracted from medical records as classification criteria. This convolutional neural network (CNN) could extract stronger features for pulse signals. Huang et al [13] developed a high-dimensional pulse classification method to improve pulse diagnosis accuracy. They extracted 71 pulse features from the time, spatial, and frequency domains to cover as much pulse information as possible.

However, most of the above methods extract structured data of pulse signals and are suitable for traditional machine learning models; however, unstructured data, such as pulse signals, contain rich information. Taking advantage of different pulse signal analysis methods and combining machine learning and deep learning methods to build pulse signals for classification models can help TCM practitioners make better pulse diagnoses and help smart wearable devices more accurately assess the human health status.

The objective of this paper was to comprehensively use machine learning and deep learning classification methods to fully exploit the information about pulse signals.

## Methods

### System Flow

**Figure 1:**
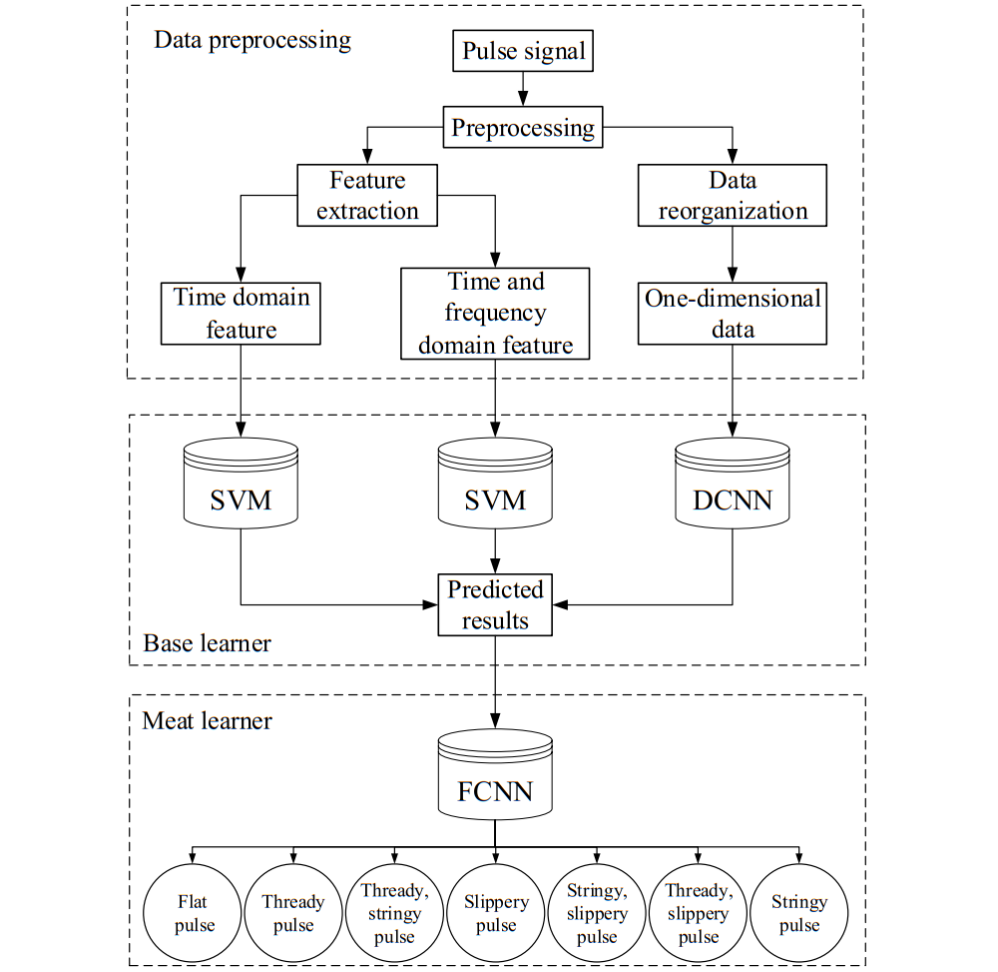
DSSN flowchart. During data preprocessing, time and frequency domain features of the pulse signal were extracted, and all pulse data were organized to the same length. Time and frequency domain features were separately trained by an SVM to obtain prediction results. One-dimensional data using the DCNN were used to obtain prediction results. Finally, the pulse-type prediction results of the three methods were integrated by an FCNN. SVM: support vector machine; DCNN: deep convolutional neural network; DSSN: DCNN- and SVM-based stacking network; FCNN: fully connected neural network.

A deep convolutional neural network (DCNN)- and SVM-based stacking network (DSSN) selected suitable algorithms to build classification models based on the structured and unstructured data extracted by different analysis methods and integrated them using the stacking method; the overall architecture is shown in Figure 1. First, the pulse signal was preprocessed to extract the feature parameters in time and frequency domains, and the data were reorganized for the pulse signal to prepare the data for the training of the base learners. An SVM and a DCNN were selected as the base learners to build the classification models corresponding to the three analysis methods, and the output results were combined together to form a new data set. Finally, the newly generated data set was used to train the meta-learner so as to build a DSSN pulse signal integrated classification model.

### Data

The experimental data in this paper were provided by the Four Diagnostic Information Comprehensive Research Laboratory of Shanghai University of Traditional Chinese Medicine, which included 7 types of pulse data (4 single pulses and 3 compound pulses), with a total sample size of 1812 cases; the specific pulse types and numbers are shown in Table 1. The acquisition device was a Z-BOX I pulse acquisition instrument with a sampling frequency of 720 Hz to acquire the pulse signals at the optimal pulse-taking pressure with an acquisition time of 60 s. Two or more TCM experts classified the collected pulse signals using their experience, and the pulse type was determined only when the majority of the experts agreed on the classification.

**Table 1:** Types of pulse signals and data size. There were 4 single pulses and 3 compound pulses. A total of 1812 cases were collected. After balancing the sample data, the total number of samples reached 4355.

Pulse type code	Name of pulse type	Sample size (n)	Balanced sample size (n)
1	Slippery pulse	221	637
2	Flat pulse	96	649
3	Thready pulse	92	607
4	Stringy pulse	657	630
5	Thready, slippery pulse	202	583
6	Thready, stringy pulse	325	614
7	Stringy, slippery pulse	219	635

**Figure 2:**
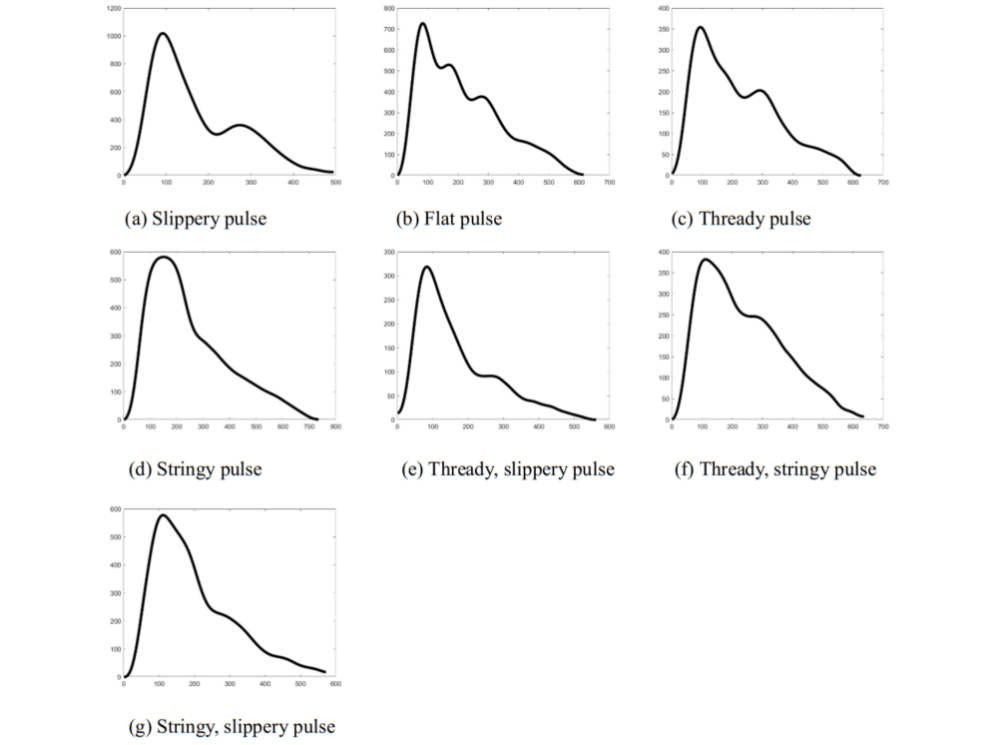
Seven types of pulse. Single pulses had four types: slippery, flat, thready, and stringy. Compound pulses had three types: thready slippery, thready stringy, and stringy slippery.

The experimental data were first preprocessed, the samples were filtered and noise reduced, single-cycle segmentation of the pulse signal was performed, and the average single cycle was taken to represent the pulse signal; seven types of pulse data are shown in Figure 2. These data set also suffered from sample imbalance, and the synthetic minority oversampling technique (SMOTE) algorithm was used to equalize the data set. For data sets with sample imbalance, the basic approaches are oversampling and downsampling (ie, copying a small number of samples and removing a larger number of samples), but both pose problems: copying samples can easily make the model overfit, while removing samples can lead to a smaller number of samples. To solve such problems, the sampling SMOTE algorithm, which synthesizes new data, can compensate for the sample imbalance, while trying to avoid overfitting [14].

The number of samples in the equalized data set was 4355 in total after removing samples with failed acquisition and obvious errors in the waveform in the data set. The number of samples in each category is shown in Table 1. The SMOTE parameters were set as *k* = 5; sample multiplicity *N* = 7 for the flat, thready pulse; sample multiplicity N = 3 for the slippery, thready and slippery, and stringy and slippery pulses; and sample multiplicity *N* = 2 for the thready, stringy pulse.

### Time Domain Feature Extraction

**Figure 3:**
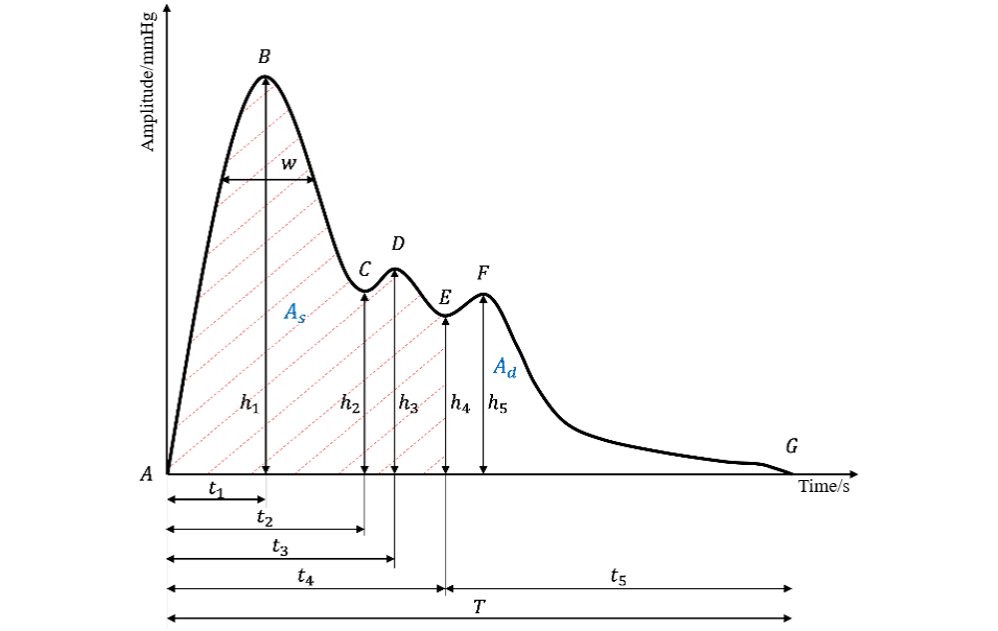
Basic information about a single-cycle pulse signal. The amplitude information about the pulse signal is represented vertically, and the time information is represented horizontally.

The time domain analysis method focuses on the waveform of the pulse signal in a typical cycle [15], defining the characteristic points with physiological and pathological significance and then extracting the corresponding characteristic parameters. In a single-cycle pulse signal, the peak and trough points of the waveform have certain physiological significance, and there are seven main feature points, including the start and end points, as shown in the marked A-G in Figure 3. The reference points and significance of the pulse waveform are shown in Table 2. The time domain feature extraction could be automatically performed using the algorithm of signal processing.

**Table 2:** Physiological significance of pulse signal reference points. These points include the start and end points of the pulse signal, as well as the extreme points of the pulse signal, all of which reflect to some extent the physiological information about the human body.

Reference point		Meaning
**Systolic waveform**		
	A	Start point
	B	Main wave crest
	C	Main wave gap
	D	Rebattling the former wave crest
**Diastolic waveform**		
	E	Descending the middle gorge
	F	Rebattling the wave
	G	End point

In this paper, a total of 23 time domain features of the pulse signal were extracted, including 1 slope feature, 2 area features, 6 amplitude features, 6 time features, and 8 proportional features; the feature parameters and their specific meanings are shown in Table 3. When extracting the pulse waveform parameters, the ratio between different amplitudes was added as a feature in order to better reflect the waveform characteristics, such as *h_2_*/*h_1_* and *h_4_*/*h_1_*, because of the large differences between different pulse waveforms. Similarly, the ratio between time parameters was increased to better distinguish between different types of pulse signals.

**Table 3:** Time domain features of the pulse signal. These features include 1 slope feature, 2 area features, 6 magnitude features, 6 time features, and 8 proportional features.

Feature type	Feature parameter	Feature name
Slope	*k*	Main wave slope
Area	*A*_s_	Systolic area
	*A*_d_	Diastolic area
Magnitude	*h*_1_	Main wave amplitude
	*h*_2_	Main wave gorge amplitude
	*h*_3_	Wave front dicrotic amplitude
	*h*_4_	Dicrotic notch amplitude
	*h*_5_	Dicrotic wave amplitude
	*w*	1/3 pulse width
Time	*t*_1_	Main wave phase
	*t*_2_	Main wave gorge phase
	*t*_3_	Wave front dicrotic phase
	*t*_4_	Dicrotic notch phase
	*t*_5_	Dicrotic wave phase
	*T*	Pulse cycle
Proportion	*t*_1_/*T*	Time ratio
	*t*_1_/*t*_4_	Time ratio
	*t*_5_/*t*_4_	Time ratio
	*w*/T	Pulse width cycle ratio
	*h*_2_/*h*_1_	Main wave gorge main Wave amplitude ratio
	*h*_4_/*h*_1_	Dicrotic notch main wave amplitude ratio
	*h*_5_/*h*_1_	Dicrotic wave main wave amplitude ratio
	*A*_s_/*A*_d_	Systolic:diastolic area ratio

### Time and Frequency Domain Feature Extraction

**Figure 4:**
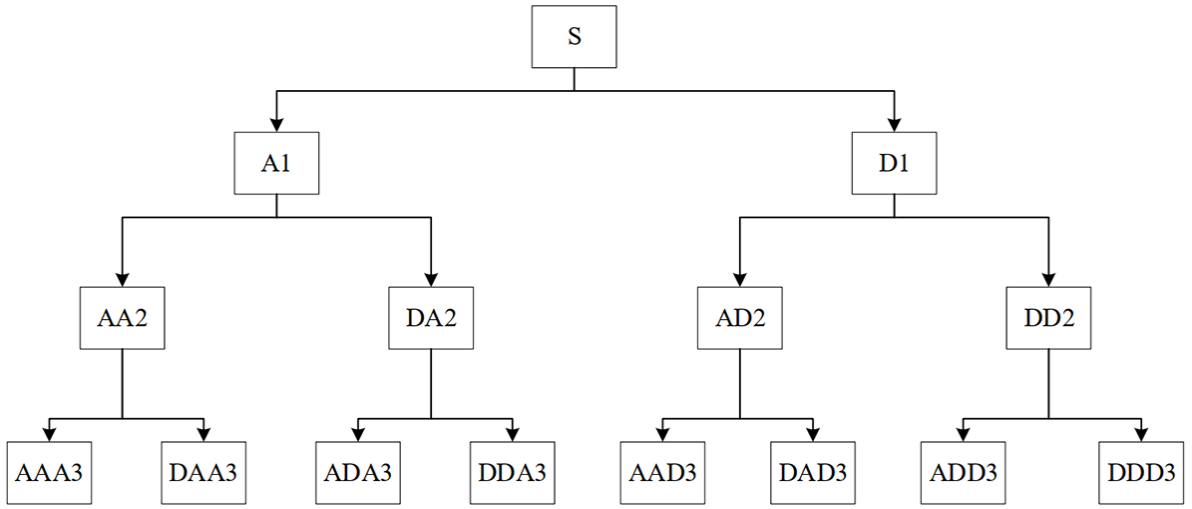
Schematic diagram of wavelet packet decomposition.

Wavelet packet analysis is an effective signal analysis method that uses different wavelet bases for signal decomposition and has a greater advantage in analyzing nonstationary signals. As a time frequency analysis method, wavelet packet analysis can zoom in on both time domain information and frequency domain information and has excellent time frequency local analysis capability. At present, it is applied to the analysis and identification of pulse signals, with good results [16]. As shown in Figure 4 as a schematic diagram of wavelet packet decomposition, wavelet packet analysis further decomposed the high-frequency band, while decomposing the low-frequency band, which improved the time-frequency resolution of the pulse signal. The low-frequency profile and high-frequency details of the wavelet packet decomposition, *u*_n_(*t*), at different frequencies is defined as follows:



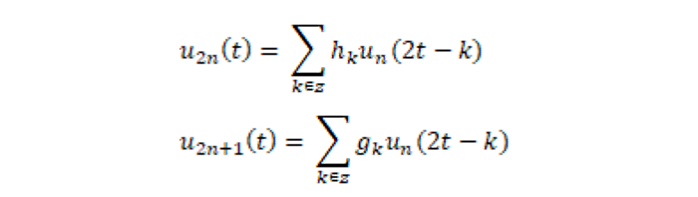



where *t* is time, *k* is the time translation factor, and *g*_k_ and *h*_k_ have an orthogonal relationship, that is, *g*_k_ = (–1)*^k^h*_1–k_. Defining the number of coefficients in the *j* layer as *n*_j_, the energy of the *j*-th layer is as follows:



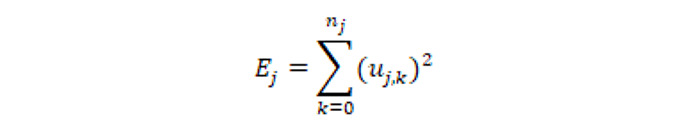



The sampling frequency of the pulse signal was 720 Hz, and the energy of the pulse was mainly concentrated in the frequency band within 10 Hz. Therefore, the energy characteristics of the pulse signal in different frequency bands were extracted by 8-layer wavelet packet decomposition. For wavelet feature extraction of biological signals, sym8 outperforms Haar, dB2, and dB4 in terms of the performance index, specificity, sensitivity, accuracy, time delay, and quality assessment of wavelets [17]. A sym8 wavelet has better regularity and symmetry, which can reduce the phase distortion caused by the calculation. Combined with the above analysis, this paper used a sym8 wavelet to decompose the pulse signal by 8-layer wavelet packets to obtain 256-dimensional energy features. The time and frequency domain features could be automatically extracted by the algorithm of signal processing.

### Classification Methods

The pulse signal features extracted by using time domain and time frequency domain analyses are structured data, and unstructured data are obtained by using data reorganization to obtain one-dimensional data of the pulse signal. For structured data, a machine learning algorithm was applied; for unstructured data, a deep learning algorithm was used to train the classification model. Finally, all results were integrated using the stacking method. The algorithms used were as follows:

1. SVM: As a base learner, this method was used to train time domain and time frequency domain data. The penalty parameter C was set as 2.0, the kernel was set as rbf, and gamma was set as 3.0. The inputs to the SVM classifier were time domain features and time frequency domain features of the pulse. The outputs were the classification results of seven pulse types.

2. DCNN: As a base learner, the pulse signal whose length was 800 was the input to the DCNN classifier, and the outputs were the classification results of seven pulse types. The network used in this paper had three convolutional layers. The first layer was set as filters = 5 and kernel size = 11. The second layer was set as filters = 25 and kernel size = 9. The third layer was set as filters = 100 and kernel size = 10. The dense layer was set as units = 7 and activation = softmax.

3. FCNN: As a meta-learner, the input was results of the three base learners and the labels were the same as raw data. The outputs were classification results of the seven pulse types after stacking. The network had four dense layers. The first and second layers were set as units = 1024 and activation = relu. The third layer was set as units = 512 and activation = relu. The last layer was set as units = 7 and activation = softmax.

In this paper, deep neural networks, including the FCNN, were implemented by TensorFlow (Google) as the back-end Keras framework, the loss function was chosen as cross-entropy, the batch size of the DCNN was set to 8, the batch size of the FCNN was set to 32, the number of iterations was 1000, the initial learning rate was 0.001, the stochastic gradient descent (SGD) optimization algorithm was used, the momentum was 0.9, the weight recession was 0.0001, the dropout parameter was 0.5, and the ratio between the training set, validation set, and test set was 6:2:2. To avoid the overfitting phenomenon caused by network training, the early stop strategy was used. When the loss of the neural network on the validation set did not decrease within 10 cycles, the training was stopped and the model with the smallest loss on the validation set was selected as the final training result. The CPU used for the experiments was an Intel Core i7-8700K with 32 GB of memory and an NVIDIA Tesla V100 GPU graphics card.

The data set in this paper contained multiple pulse categories, so it was necessary to combine the classification results of each category for judgment. In this paper, macroaverage was used as the judging index, and the average accuracy and average recall were calculated as follows:



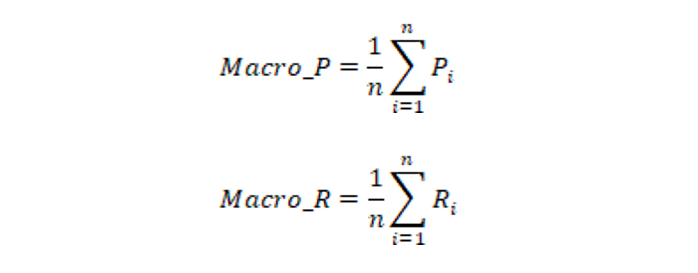



## Results

### SVM Experimental Results

In training the SVM model, the radial basis function (RBF) was chosen as the kernel function, in which the main parameters included the penalty coefficient C and gamma. c indicated the degree of acceptance of error; the larger the value of C, the less the classification error was allowed to occur during training, and the selection of appropriate C could suppress the overfitting phenomenon of the model. Here, gamma was a parameter of the RBF, which was used to adjust the range of action of the model support vector.

To find the best parameter that made the best classification of the model, this paper used the grid search method to determine optimal parameter values. When training the classification model with time domain features and time frequency domain features, the parameter range of C was set to 1-50, with a step size of 1; the parameter range of gamma was 1-50, with a step size of 0.5; and default values were used for the rest of the parameters. The results of the optimal classification model are shown in Table 4.

**Table 4:** Classification results of time and time frequency domain features with an SVM^a^. The average accuracy rate is the percentage of all pulse type classifications that are correct. The average recall rate is the ratio of the correct pulse type in the classification result to the pulse type in the sample. Accuracy is the average of the accuracy of each of the 7 pulse types.

Classification model	Average accuracy rate (%)	Average recall rate (%)	Accuracy (%)
Time domain feature+SVM	79.2	76.2	76.1
Time and frequency domain feature+SVM	74.6	72.9	72.8

^a^SVM: support vector machine.

As can be seen from Table 4, the time domain classification model had a higher accuracy than the time frequency domain classification model with the same classifier, reaching 76.1%, which was 3.3% higher than the time frequency domain classification model. Meanwhile, the flat accuracy rate and the average recall rate of the time domain classification model were 79.2% and 76.2%, respectively, which were higher than those of the time frequency domain classification model.

### DCNN Experimental Results

To verify the classification performance of the DCNN used in this paper, two neural networks, Visual Geometry Group (VGG)-11 and VGG-16, were selected for comparison experiments. In the experiments, VGG-11 adopted the standard network structure and VGG-16 adopted the improved network structure. The initial learning rate of both CNNs was 0.0001, and the rest of the parameters were the same as those of the DCNN. The experimental results are shown in Table 5.

**Table 5:** Classification results of different CNN^a^ structures. The average accuracy rate is the percentage of all pulse type classifications that are correct. The average recall rate is the ratio of the correct pulse type in the classification result to the pulse type in the sample. Accuracy is the average of the accuracy of each of the 7 pulse types.

Classification model	Average accuracy rate (%)	Average recall rate (%)	Accuracy (%)
VGG^b^-11	74.4	74.7	74.7
VGG-16	77.3	77.3	77.4
DCNN^c^	79.1	78.9	79.1

^a^CNN: convolutional neural network.

^b^VGG: Visual Geometry Group.

^c^DCNN: deep convolutional neural network.

As can be seen from Table 5, the DCNN had the highest accuracy rate, which was 4.4%, and 1.7% higher compared with VGG-11 and VGG-16, respectively. In the average accuracy and average recall, the VGG-11 network had the lowest rates among the three models, which was 4.7% and 1.8% lower compared with the highest DCNN, respectively.

### DSSN Experimental Results

To objectively evaluate the effectiveness of the model DSSN proposed in this paper, the base learners of the models were compared, and the experimental results are shown in Table 6.

**Table 6:** Classification results of different algorithms. The average accuracy rate is the percentage of all pulse type classifications that are correct. The average recall rate is the ratio of the correct pulse type in the classification result to the pulse type in the sample. Accuracy is the average of the accuracy of each of the 7 pulse types.

Classification model	Average accuracy rate (%)	Average recall rate (%)	Accuracy (%)
Time and frequency domain feature+SVM^a^	74.6	72.9	72.8
Time domain feature+SVM	79.2	76.2	76.1
DCNN^b^	79.1	78.9	79.1
DSSN^c^	83.2	82.9	83.3

^a^SVM: support vector machine.

^b^DCNN: deep convolutional neural network.

^c^DSSN: DCNN- and SVM-based stacking network.

As can be seen from Table 6, the DSSN model had the highest classification accuracy among the five methods, reaching 83.3%. In the average accuracy and average recall, the DSSN model rates improved by 8.6% and 10%, respectively, compared with the lowest time frequency domain feature model, and 4.1% and 4%, respectively, compared with the remaining highest DCNN model.

## Discussion

Using machine learning or deep learning alone for pulse classification is not effective, but integrating both for learning can improve the classification accuracy of pulse signals. At the same time, existing research results on interpretable features of pulse signals can be absorbed and deep learning algorithms developed by technology can be used to further explore the information carried by pulse signals.

### Comparison of Seven Pulse Type Classification Results Using an SVM

**Figure 5:**
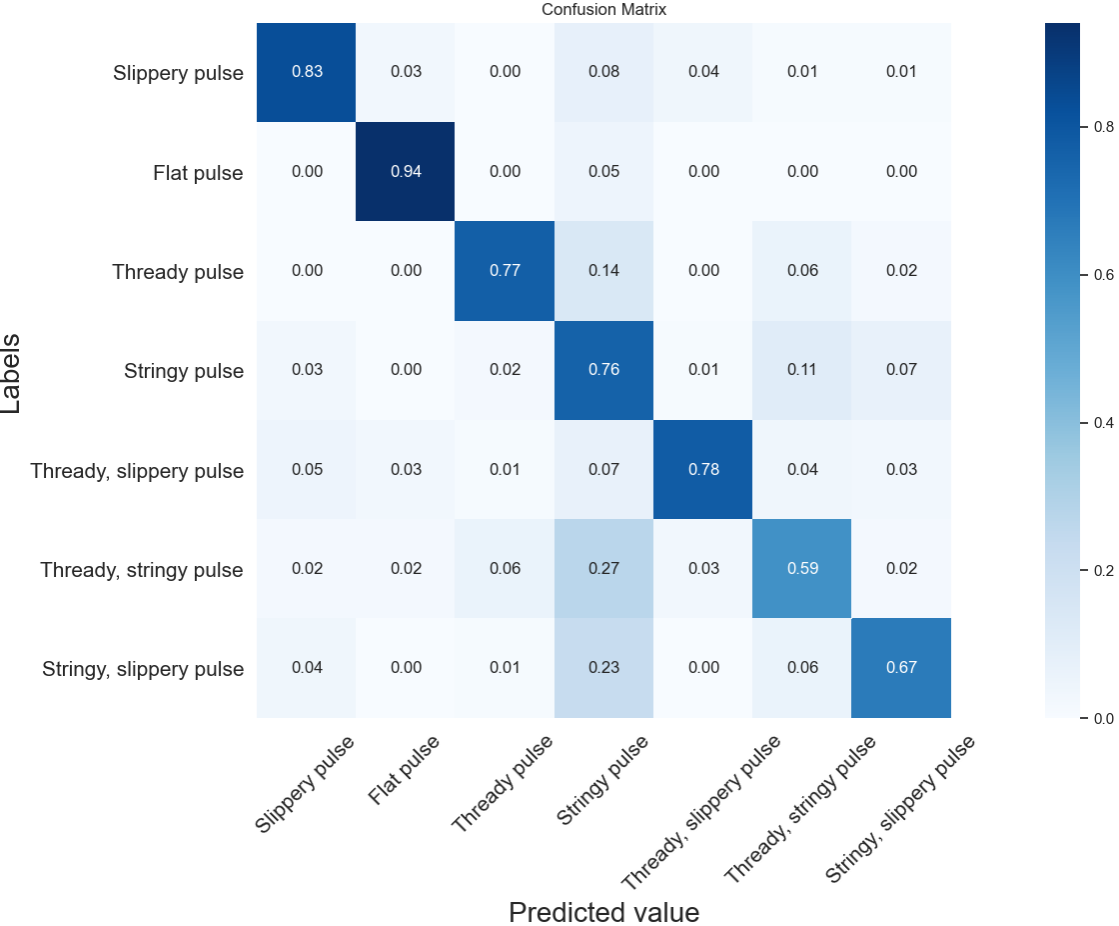
Confusion matrix of time domain features by SVM classification. SVM: support vector machine.

**Figure 6:**
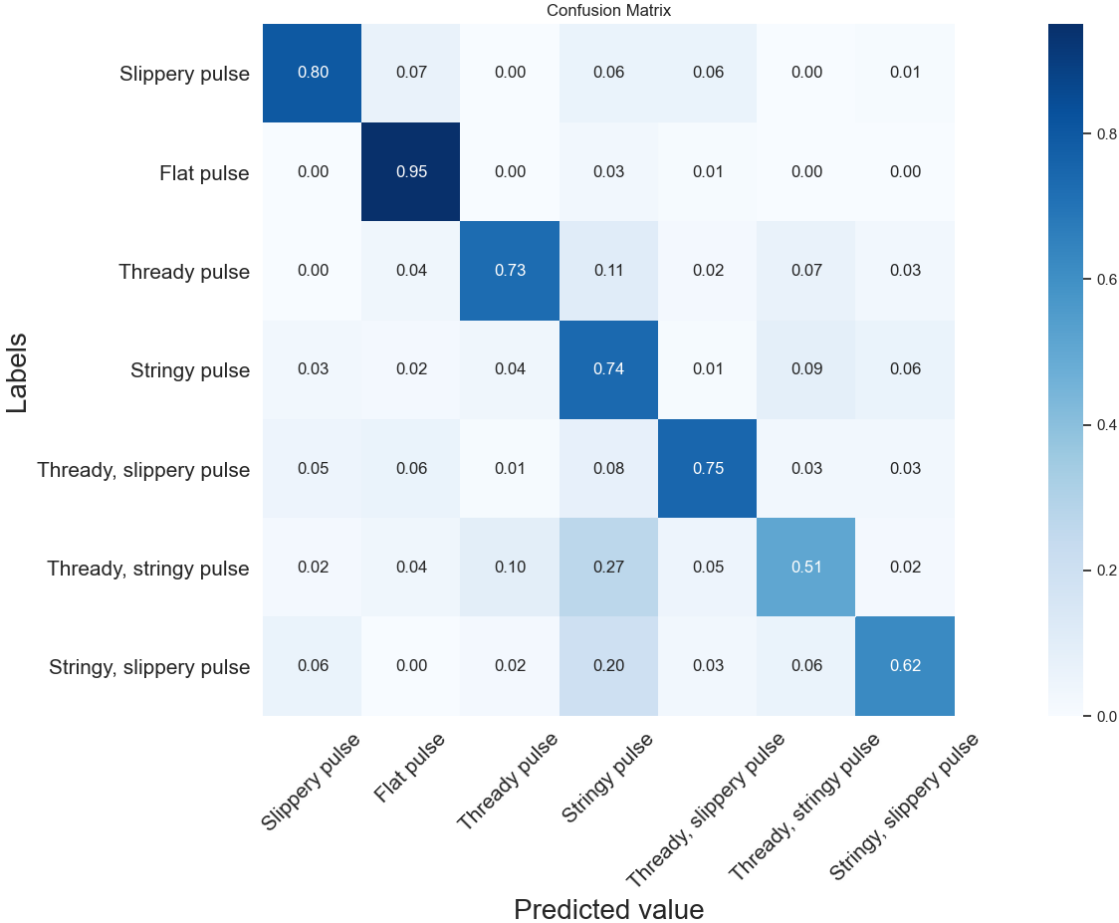
Confusion matrix of time and frequency domain features by SVM classification. SVM: support vector machine.

As can be seen from Figures 5 and 6, the recognition accuracy of the time domain classification model was higher than that of the time frequency domain classification model in the slippery, thready slippery, thready, thready stringy, stringy slippery, and stringy pulses, reaching 83%, 78%, 77%, 59%, 67%, and 76%, respectively, which was 2%-8% higher compared with the time frequency domain classification model. However, the time frequency domain classification model had the highest recognition rate of 95% for the flat pulse, which was 1% higher compared with the time domain classification model.

Overall, the time domain classification model was slightly better than the time frequency domain classification model. With the exception of the thready, stringy pulse, the accuracy of the recognition of the remaining pulse types was not significantly different. It can also be seen from the figure that the thready, stringy pulse and the stringy, slippery pulse were the pulse types with the lowest classification accuracy, and most of them were incorrectly classified as stringy pulses. In the time domain classification model, the recognition error rates were 27% and 23% for the thready, stringy and the stringy, slippery pulses, respectively; in the time frequency domain classification model, the recognition error rates were 27% and 20%, respectively. The reason for this situation might be that both the thready, stringy pulse and the stringy, slippery pulse have the characteristics of a stringy pulse, and it is difficult for the classifier to accurately determine their pulse types, leading to misclassification.

Because of the large number of time domain features extracted in this paper, only some features were selected for statistical analysis, as shown in Table 7. Among the seven types of pulse data, the main wave slope *k* of the slippery pulse was the largest, and the main wave amplitude *h*_1_ was only second to the stringy, slippery pulse, which showed the characteristics of the high and steep main wave of the slippery pulse. The one-third pulse width *w* and pulse width period ratio *w*/*T* of the stringy pulse were the largest among the seven types of pulses, which showed the waveform characteristics of the wide main wave of the stringy pulse. The main wave amplitude *h*_1_ of the stringy, slippery pulse was the largest, the main wave slope *k* was only lower than that of the slippery pulse, and the one-third pulse width *w* and pulse width period ratio *w*/*T* were larger, which showed that the stringy, slippery pulse had the waveform characteristics of both the stringy pulse and the slippery pulse, which was consistent with the characteristics of both pulses.

**Table 7:** Statistical analysis results of some time domain features. Feature parameters were h1, k, w, and w/T for 7 pulse types.

Feature parameters	Slippery pulse	Flat pulse	Thready, slippery pulse	Thready pulse	Thready, stringy pulse	Stringy, slippery pulse	Stringy pulse
*h*_1_	610.701 ± 206.724	514.706 ± 121.698	356.757 ± 93.869	400.857 ± 139.692	407.703 ± 157.803	636.707 ± 227.226	592.404 ± 228.413
*k*	6.609 ± 2.241	5.886 ± 1.394	3.912 ± 1.002	3.662 ± 1.161	3.957 ± 1.468	6.329 ± 2.420	5.919 ± 2.543
*w*	0.126 ± 0.018	0.143 ± 0.031	0.140 ± 0.030	0.199 ± 0.034	0.205 ± 0.043	0.176 ± 0.036	0.207 ± 0.044
*w*/*T*	0.162 ± 0.023	0.165 ± 0.036	0.185 ± 0.040	0.214 ± 0.035	0.247 ± 0.038	0.220 ± 0.033	0.245 ± 0.038

The time domain features not only reflect the waveform characteristics of the pulse signal but also have certain physiological and pathological significance. The amplitude of the main wave, *h*_1_, reflects the ejection function of the left ventricle and the compliance of the aorta; the amplitude of the main wave isthmus, *h_2_*, has the same significance as the amplitude of the pre-repulse wave, *h*_3_, and the sclerosis of blood vessels or the increase in peripheral resistance leads to an increase in the amplitudes *h_2_* and *h*_3_. The amplitude of the descending isthmus, *h*_4_, reflects the magnitude of peripheral resistance. The magnitude of the repulse wave, *h_5_*, reflects the level of compliance of the aorta. The magnitude of phase *t*_1_ reflects the rapidity of the left ventricular ejection time; the magnitudes of descending isthmus phase *t*_4_ and repulse wave phase *t*_5_ reflect the length of the systolic and diastolic phases of the left ventricle, respectively. The pulse period *T* indicates one cycle of the pulse, corresponding to one cardiac cycle of the left ventricle. The ratio of the descending isthmus main wave amplitude, *h*_4_/*h*_1_, reflects the level of peripheral resistance; the ratio of the repulse wave main wave amplitude, *h*_5_/*h*_1_, reflects the vascular compliance. The time ratio *t*_1_/*T* reflects the rate of the cardiac ejection function, which increases when the rate decreases [18]. Wavelet packet analysis is used in the time frequency domain analysis to extract the energy magnitude of the pulse signal in different frequency bands, and its distribution reflects the elastic changes in the blood vessels [19], which also has some physiological significance.

In the classification algorithm, the SVM uses the kernel RBF to map the feature parameters into a high-dimensional space to provide better differentiability between different classes. The segmentation hyperplane is trained under this space to give the classification model the ability to recognize different pulse types. Thus, the time domain and time frequency domain features characterize the pulse signal from different perspectives, which can be combined with the SVM algorithm to obtain better classification results.

### Comparison of Seven Pulse Type Classification Results Using VGG-11, VGG-16, and the DCNN

**Figure 7:**
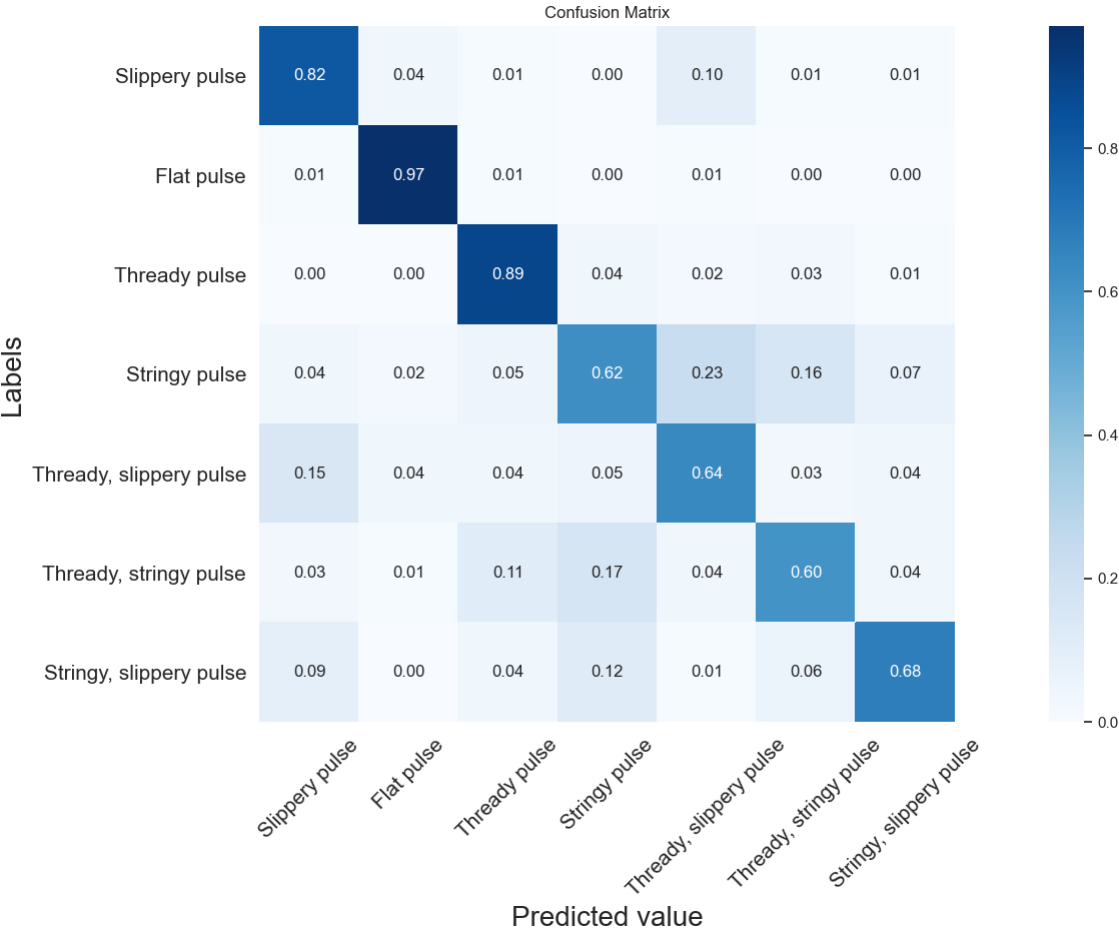
Confusion matrix of the seven types of pulses by VGG-11. The diagonal elements of the matrix indicate the prediction accuracy of different types of pulses. VGG: Visual Geometry Group.

**Figure 8:**
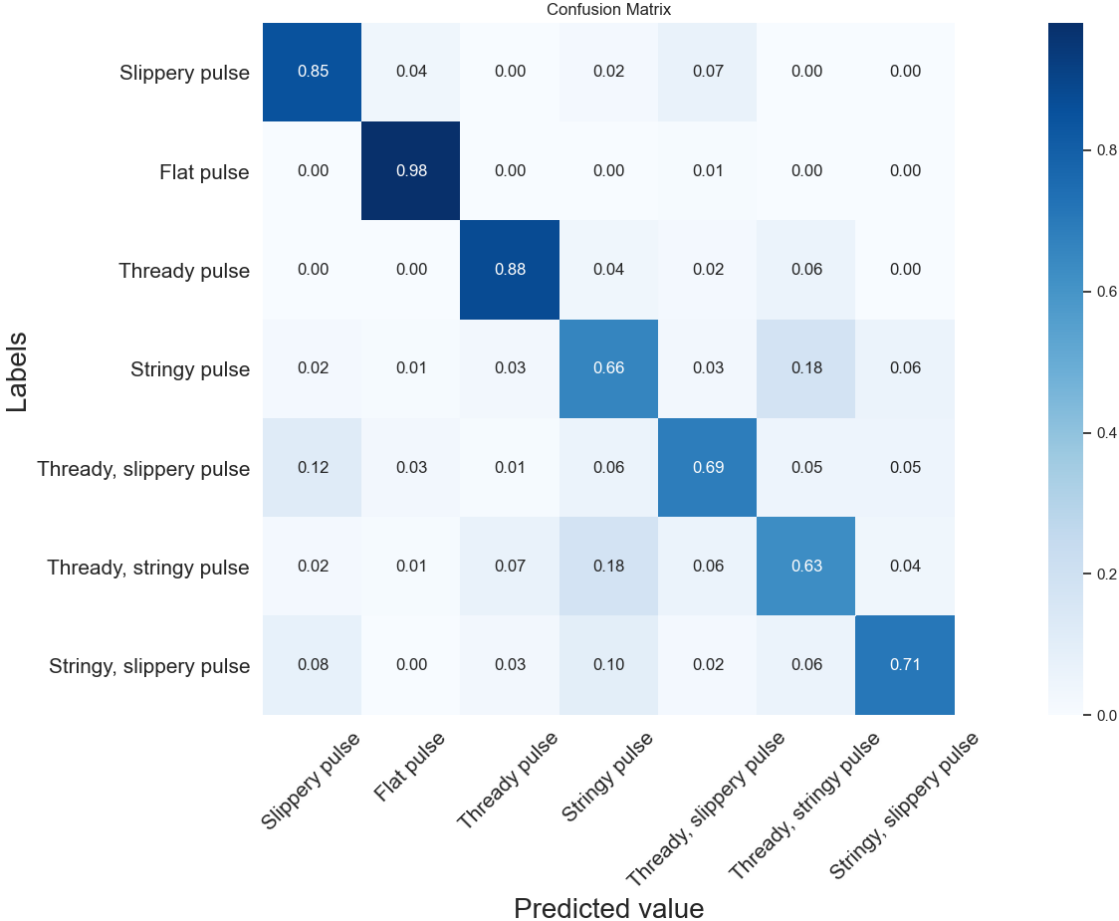
Confusion matrix of the seven types of pulses by VGG-16. The diagonal elements of the matrix indicate the prediction accuracy of different types of pulses. VGG: Visual Geometry Group.

**Figure 9:**
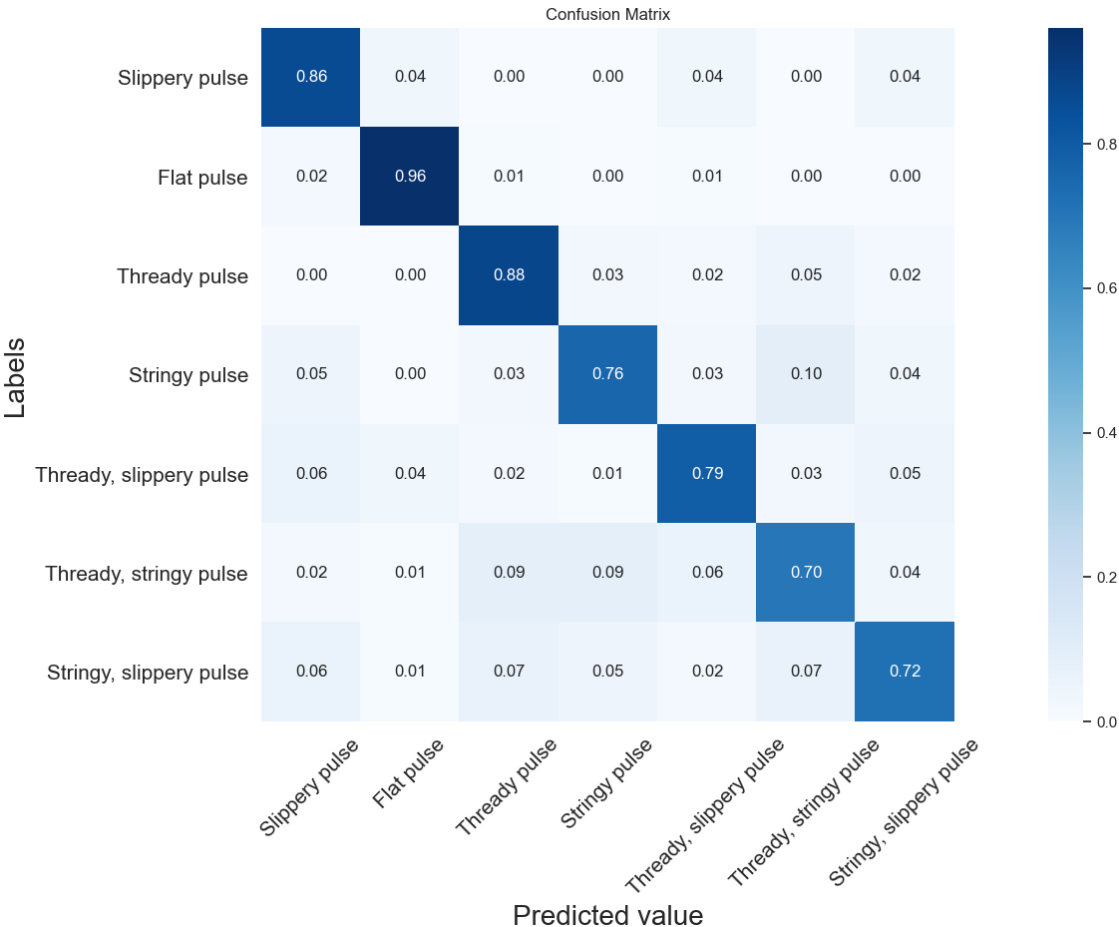
Confusion matrix of the seven types of pulses by the DCNN. The diagonal elements of the matrix indicate the prediction accuracy of different types of pulses. DCNN: deep convolutional neural network.

Figures 7-9 show confusion matrix plots of the three neural networks on the seven types of pulse data sets. As can be seen from the figures, the recognition accuracy of the DCNN improved in different degrees compared with VGG-11 and VGG-16 for the all categories of pulses except the flat pulse. Compared with VGG-11, the DCNN had a 6% higher recognition rate in the fine slippery pulse and the stringy, slippery pulse and a 5% higher recognition rate in the slippery pulse and the stringy pulse. Compared with VGG-16, the recognition rate was 1%-4% higher in all categories of pulses except the flat pulse. In the flat pulse recognition rate, VGG-16 had the highest accuracy of 98%, which was 3% higher than that of the DCNN. The recognition rate of the compound pulse was low overall, where most of the misclassified samples were classified as single-pulse types contained in the compound pulse, which might be due to the fact that the compound pulse had features that made up two of its single-pulse types, resulting in the classifier being unable to correctly identify its pulse type, similar to the results of the time-domain and time frequency-domain classification models. Overall, the DCNN had better classification performance compared with VGG-11 and VGG-16.

### Comparison of Seven Pulse Type Classification Results Using a DSSN

**Figure 10:**
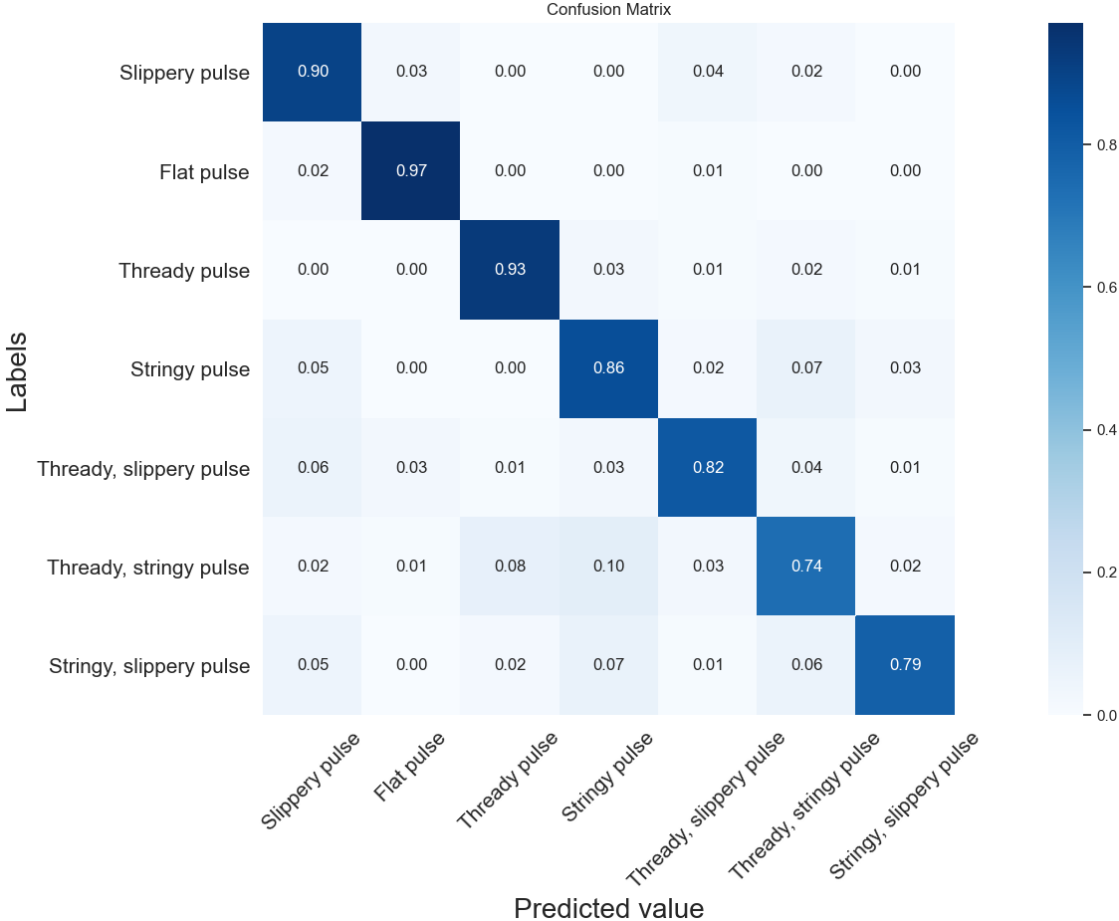
Confusion matrix of the DSSN. DSSN: deep convolutional neural network (DCNN)- and support vector machine (SVM)-based stacking network.

It can be seen from Figure 10 that compared with the three base learners, the DSSN model improved the recognition rate in all seven pulse categories to varying degrees, with 3%, 2%, 4%, 1%, 10%, 5%, and 10% improvement compared to the highest recognition rate in each category of the base learners, respectively. Among them, the thready, stringy pulse, the stringy, slippery pulse, and the stringy pulse had the highest improvement effect, which reduced the recognition error rate to a greater extent. In addition, the DSSN model had higher recognition accuracy in each category than the DCNN model. Among them, the recognition rate of the stringy pulse was 10% higher compared with the DCNN model, and the remaining pulse types improved by 1%-7%. Therefore, the DSSN could integrate the advantages of multiple base learners and thus effectively improve the recognition accuracy of the model. Compared with existing pulse signal classification models, the DSSN also had better classification recognition results.

Although pulse types are clearly described in TCM textbooks, different TCM practitioners often interpret pulse diagnoses differently, depending on their own experience and understanding of pulses [20]. Even the same TCM practitioner may make different diagnoses for similar pulse characteristics in different circumstances. Using machine learning and deep learning methods for pulse classification can help TCM practitioners make better pulse diagnoses and improve the objectivity of the results. Using the DSSN method, the recognition accuracy for compound pulses can reach more than 70%, while for single pulses, the recognition accuracy is above 85%, and the best one can reach 97%. If the experimental samples can be enriched and the balance of samples can be improved, the accuracy of pulse classification can be further improved, which is promising for application in wearable devices.

### Limitations and Conclusion

The existing work on pulse classification was mainly performed by machine learning or deep learning. In this paper, pulse classification was performed by a machine learning model (SVM) and a deep learning network (DCNN), but the results were not good. Through the DSSN method, the classification results of machine learning and deep learning were ensembled to obtain more accurate pulse type prediction results. Practitioners of TCM can use this method to assist in the diagnosis of TCM pulses, thus avoiding the uncertainty caused by subjectivity. Wearable devices can also use this method to determine the type of pulse of the user and thus predict the health status of the user, which is also relevant for the prevention of some diseases. At the same time, there were some areas for improvement in this experiment. First, the sample data of the pulse should be as large as possible, which will help improve the accuracy rate to some extent. Second, the diagnosis of the type of pulse signal collected should be integrated with the diagnosis results of several TCM experts, which can further improve the objectivity of the data.

A large number of original pulse wave studies have yielded many interpretable features, and many research results have been obtained for pulse wave classification. If only deep learning is used for classification, it will be difficult to use the results of previous research. Deep learning feature engineering and structured features reflect different pulse feature information that can complement each other. Therefore, based on the original pulse analysis, TCM scholars can combine the advantages of deep learning algorithms developed by technology to construct integrated classifiers that can provide better classification results by making full use of the information obtained by deep learning feature engineering and artificially constructed features.

## Abbreviations

CNN: convolutional neural network
CVD: cardiovascular disease
DCNN: deep convolutional neural network
DSSN: DCNN- and SVM-based stacking network
ERP: edit distance with real penalty
FCNN: fully connected neural network
KNN: k-nearest neighbor
RBF: radial basis function
SGD: stochastic gradient descent
SMOTE: synthetic minority oversampling technique
SVM: support vector machine
TCM: traditional Chinese medicine
VGG: Visual Geometry Group

## References

[1] O'Rourke M, Pauca A, Jiang X (2001). Pulse wave analysis. Br J Clin Pharmacol.

[2] Korpas D, Hálek J, Dolezal L (2009). Parameters describing the pulse wave. Physiol Res.

[3] Safar ME, Levy BI, Struijker-Boudier H (2003). Current perspectives on arterial stiffness and pulse pressure in hypertension and cardiovascular diseases. Circulation.

[4] Yamashina A, Tomiyama H, Arai T, Hirose Ken-ichi, Koji Y, Hirayama Y, Yamamoto Y, Hori S (2003). Brachial-ankle pulse wave velocity as a marker of atherosclerotic vascular damage and cardiovascular risk. Hypertens Res.

[5] Cohn Jn, Finkelstein S, McVeigh G, Morgan D, LeMay L, Robinson J, Mock J (1995). Noninvasive pulse wave analysis for the early detection of vascular disease. Hypertension.

[6] Anson CYT (2012). Review of traditional Chinese medicine pulse diagnosis quantification. Complement Ther Contemp Healthc.

[7] Lee J-Y, Jang M, Shin S-H (2017). Study on the depth, rate, shape, and strength of pulse with cardiovascular simulator. Evid Based Complement Alternat Med.

[8] Xu LS, Meng MQ, Wang KQ (2007). Pulse image recognition using fuzzy neural network. Annu Int Conf IEEE Eng Med Biol Soc.

[9] Xu L, Zhang D, Wang K, Wang L (2006). Arrhythmic pulses detection using Lempel-Ziv complexity analysis. EURASIP J Adv Signal Process.

[10] Zhang D, Zuo W, Zhang D, Zhang H, Li N (2010). Classification of pulse waveforms using edit distance with real penalty. EURASIP J Adv Signal Process.

[11] Garmaev BZ, Boronoev VV, Naguslaeva IV, Ompokov VD (2019). Classification of pulse waves based on cluster analysis of time parameters. J Phys: Conf Ser.

[12] Li G, Watanabe K, Anzai H, Song X, Qiao A, Ohta M (2019). Pulse-wave-pattern classification with a convolutional neural network. Sci Rep.

[13] Huang C-H, Wang Y-M, Smith S (2020). Using high-dimensional features for high-accuracy pulse diagnosis. Math Biosci Eng.

[14] Fernandez A, Garcia S, Herrera F, Chawla Nv (2018). SMOTE for learning from imbalanced data: progress and challenges, marking the 15-year anniversary. jair.

[15] Chen Y-H, Yang CC, Cao QF, Li BT, Shang YS (2006). The comparison of some time-frequency analysis methods. Prog Geophys.

[16] Guo Q, Wang K, Zhang D (2008). A wavelet packet based pulse waveform analysis for cholecystitis and nephrotic syndrome diagnosis.

[17] Deepa R, Rajaguru H, Ganesh Babu C (2020). Analysis on wavelet feature and Softmax discriminant classifier for the detection of epilepsy.

[18] Zhang W, Zhang Y, Zhang S (2008). Application on wavelet and neural network in the analysis and pattern recognition of the manifestation of the pulse for detection of cerebrovascular disease. Shanghai Biomed Eng.

[19] Hu X, Zhu H, Xu J (2014). Wrist pulse signals analysis based on deep convolutional neural networks.

[20] Alice LYL, Guan B, Chen C, Chan H, Kong K, Li W, Shen J (2021). Artificial intelligence meets traditional Chinese medicine: a bridge to opening the magic box of sphygmopalpation for pulse pattern recognition. Digital Chin Med.

